# Tactile Sensory Dysfunction in Children with ADHD

**DOI:** 10.3233/BEN-2008-0221

**Published:** 2009-07-29

**Authors:** Ahmad Ghanizadeh

**Affiliations:** ^1^Department of PsychiatryShiraz University of Medical SciencesHafez HospitalShirazIran; ^2^Research Center for Psychiatry and Behavioral SciencesShiraz University of Medical SciencesHafez HospitalShirazIran

**Keywords:** ADHD, ODD, Tactile sensory dysfunction, children, gender, checklist

## Abstract

*Objectives:* While a group of children with ADHD may have normal behavioral responses to sensory stimuli, another group may be hyperreactive. The aim of this survey was studying association of tactile sensory responsivity with co-morbidity of oppositional defiant disorder (ODD) symptoms, subtypes of ADHD, and gender in children with ADHD.

*Methods:* The subjects were 81 children with ADHD from a child psychiatry clinic. The diagnoses were made according to DSM-IV diagnostic criteria. Tactile dysfunction Checklist was used to assess the three types of tactile sensory dysfunction including Hypersensitivity, hyposensitivity, and poor tactile perception and discrimination (PTPD).

*Results:* Their mean age was 8.4 (SD = 1.9) years. None of the gender, number of symptoms of ODD co-morbidity, and ADHD subtypes was as a predictor of scores of Hyposensitivity and PTPD subscales. Tactile defensiveness was not different between genders and different subtypes of ADHD.

*Conclusions:* Number of ODD symptoms in children with ADHD is a predictor in association with hypersensitivity score of tactile sensory function. Girls are no more than the boys impaired in Hypersensitivity aspect. Different subtypes of ADHD are not distinct disorders regarding to tactile sensory function.

*Abbreviations:* ADHD – attention deficit hyperactivity disorder; ODD – oppositional defiant disorder

## 1. Introduction

Tactile information or the experiences derived from receptors in the skin are relayed to the brain and processed by somesthetic or somatosensory system. This system interacts with other sensory systems. It has an important role in development of organism such as learning and cognitive functions [1].

Over responsiveness or sensory sensitivity is strongly and/or negatively reacting to sensory input which is not usually perceived as a threat by most of people. In fact, a little input elicits a response, distraction, avoidance or withdrawal behaviors, or emotional outbursts [2]. For example, a child with over responsiveness might not tolerate hair-brushing, haircuts, and/or hair washing. Under responsiveness is lack of reacting to a sensory input with the expected intensity or frequency. A high level of input is required to have a response [2].

In children with sensory avoiders, attention is hyperfocused and in sensory seekers, attention is characterized by inattention, poor impulse control, and hyperactivity. In other words, there is an overlap with symptoms of ADHD [3]. Children with ADHD showed deficits in tactile perception including temperature discrimination, the sensory-discriminative aspects of pain, and light touch. Light touch was evaluated by touching the child with a cotton wool on the skin. Light touch aspect was not significantly different between the ADHD group and control group. That study only assessed deficits in tactile perception in the ADHD children [4].

ISSN 0953-4180/08/$17.00 © 2008 – IOS Press and the authors. All rights reserved

Parental-reported measures displayed children with ADHD had greater abnormalities in sensory modulation (SMD). Levels of SMD were highly correlated with measures of psychopathology in the ADHD children. They also reported a large degree of variability in sensory processing in the children with ADHD and suggested that a group of children with ADHD may have normal behavioral responses to sensory stimuli, whereas another group may be hyperreactive and overresponsive. On the other hand, ADHD is not a homogenous group; thus, their patterns of responses to sensory stimuli might differ according to subgroups within ADHD. So, it is important to research on tactile sensory processing in subgroups of children with ADHD [5]. There is a report that females with ADHD display higher levels of tactile defensiveness than males with ADHD [5].

ADHD is a loose association of symptoms [6]. Its symptoms are very common [7]. Clinical sample of ADHD children typically have at least another one psychiatric disorder. The rate of oppositional defiant disorder (ODD) is about 59% [8,9]. Another common psychiatric disorder is separation anxiety disorder (SAD). Children with ODD have recurrent pattern of negativistic, defiant, disobedient, argumentative, anger associated, and hostile behavior toward parents and peers [10]. It is controversy whether ODD and ADHD are distinct clinical entities [11]. Studying of ODD in children with ADHD is important in order to identify possible risk factors related to the etiology, prognosis and their treatment [12].

Tactile sensory processing dysfunctions had not been studied in ADHD with co-morbidity of ODD symptoms. It was aimed to survey if co-morbidity of ODD symptoms in ADHD children would predict parental reported sensory responsivity. In addition, TD correlates with anxiety [13]. So, separation anxiety was considered as a covariant factor. Second, it was hypothesized that ADHD girls more than ADHD boys are suffering from tactile sensory dysfunction. The three types of tactile sensory dysfunction including tactile defensiveness, hyposensitivity, and poor tactile perception and discrimination are studied. All of the previous studies focused on the tactile defensiveness in ADHD children. None of them surveyed if there is any association between co-morbidity of ODD symptoms and tactile sensory dysfunction in ADHD children. Finally, studying of association of tactile sensory function problems and subtypes of ADHD was aimed.

## 2. Method

The sample consisted of 81 children (64 boys, 17 girls) with ADHD. The ADHD children were referrals of the outpatient clinic of Hafez Hospital affiliated to the Department of Child and Adolescent Psychiatry at Shiraz University of Medical Sciences. Their age range was from 6 to 14 years (mean age of 8.4 and SD of 1.9 years). They are from a broader ongoing study on sensory processing dysfunction in children with ADHD (e.g. [14]). None of the subjects were clinically estimated to be mental retarded. Children with a diagnosis of autism, epilepsy, brain disorders or known genetic disorders, such as Down syndrome or Fragile-X-syndrome were not included. All of the children were Persian. The children who were off medication for at least 4 weeks were included in the study.

ADHD was diagnosed according to Diagnostic and Statistical Manual of Mental Disorders-IV [10]. In order to make psychiatric diagnosis, the children and adolescents and their parents were interviewed by the child and adolescent psychiatrist, administering the Schedule for Affective Disorders and Schizophrenia for School Age Children (K-SADS), Farsi version [15]. The K-SADS, Farsi version, is a semi-structured psychiatric interview. Its reliability, validity, and administrative characteristics had been reported elsewhere [15]. Presence of symptoms of criteria A of DSM-IV for oppositional defiant disorder and separation anxiety disorder were surveyed.

### 2.1. *Tactile sensory dysfunction checklist*

There was not found any valid and reliable instrument to be used for assessing of the signs of tactile dysfunction that included the different three types of tactile sensory dysfunction including hypersensitivity to touch (tactile defensiveness), hyposensitivity to touch (under-responsive), and poor tactile perception and discrimination. Therefore, in the first step, a parent-report questionnaire was developed [16–18]. The checklist suggested some statements for screening tactile sensory dysfunctions. They categorized the tactile dysfunctions items into categories of hypersensitivity to touch (tactile defensiveness), hyposensitivity to touch (under-responsive), and poor tactile perception and discrimination. There is no cut-off score on the checklist serving as a clinical threshold. So, the score does not indicate tactile sensory disorder but it shows tactile sensory problem. It is not a diagnostic test and its psychometric properties were not reported before. Children with tactile defensiveness are always attending to sensory input that others ignore them or most individuals would consider harmless. Children with under-responsive may be unresponsive to some stimuli or a high level of input is necessary to have a response. The items were translated by a team of child and adolescent psychiatrist and general psychiatrists and psychologist. The items of Farsi Version were back translated. The back translated version of the items was reviewed and reconfirmed its content and face validity by the team and the final translation was fixed by consensus. To examine the feasibility of using of the questionnaire, it had been administered to parents of children in a child and adolescent psychiatric clinic. After approval of confirmed items feasibility to understand by parents and establishments of its content and face validity, it was used in the current study.

The parents provided information regarding the frequency in which the children had usually responded to the situation. The response categories were: 0 (never), 1 (occasionally), 2 (often), and 3 (always). Higher scores show more dysfunction. Oral informed consent was given by the participants.

### 2.2. Analysis

The first step of analysis was development of the checklist. Factor analysis for examining of the factor structure was used. There were three subscales. Hypersensitivity subscale included 15 items with scoring range of 0 to 45. The number of items of Hyposensitivity subscale was 8 items. Its range of scoring was 0 to 24. PTPD included 5 items. The range of scoring of PTPD was 0 to 15.

The item reliability was analyzed through Cronbach's alpha coefficients for the three subscales.

Then, three separate univariate general linear model analyses were conducted. Hypersensitivity subscale, Hyposensitivity subscale, and the poor tactile perception and discrimination subscale (PTPD) were considered as the dependent variables. Number of symptoms of ODD, separation anxiety score, gender, ADHD subtypes, and age were considered as independent variables.

## 3. Results

The three-factor solution accounted for 29.45% of the total variance in factor analysis. The items with item–total correlations of less than .30 were dropped from the checklist. The hypothesized factor structure of the 28 item questionnaire was tested using maximum likelihood factor analysis. Kaiser-Meyer-Olkin Measure of sampling adequacy was .69 and the Bartlett's Test of Sphericity was significant ($P < 0.001$). The items and subscales are indicated in the appendix.

Table 1
General linear model analysis for prediction of Tactile Hypersensitivity score

| Independent variables | df | F | Sig. |
|---|---|---|---|
| Separation anxiety score | 1 | 2.693 | 0.108 |
| Oppositional defiant behavior score | 1 | 6.356 | 0.015 |
| Age | 1 | 0.027 | 0.870 |
| Father's age | 1 | 0.490 | 0.488 |
| Father's educational level | 1 | 0.858 | 0.359 |
| Age of mother | 1 | 0.511 | 0.478 |
| Educational level of mother | 1 | 0.457 | 0.503 |
| ADHD type | 3 | 0.553 | 0.649 |
| Gender | 1 | 3.552 | 0.066 |
| Interaction of ADHD type and gender | 3 | 2.200 | 0.101 |

R Squared = 0.435 (Adjusted R Squared = 0.263).

The item reliability was analyzed through Cronbach's alpha coefficients for the three subscales. The internal consistency reliability scores were good with the Hypersensitivity subscale at 0.78, Hyposensitivity subscale at 0.76, and the poor tactile perception and discrimination subscale 0.75.

There was a correlation between Hypersensitivity subscale score and ODD score ($r = 0.4$, $P < 0.001$). Mean difference between boys and girls for Hypersensitivity subscale score was not statistically significant ($t = 0.11$, df = 78, $P = 0.9$). Univariate general linear model analyses with considering of the covariant factors indicates that ODD score in children with ADHD is associated with score of Hypersensitivity scale ($P < 0.01$). In addition, the factors of gender, age, SAD, and ADHD types were not associated with Hypersensitivity scale score (Table 1). There was a positive correlation between ODD score and Hypersensitivity subscale score ($r = 0.45$, $P < 0.001$).

Univariate general linear model analysis shows that ADHD subtypes is not associated with the Hyposensitivity scale score (Table 2). Separation anxiety score was associated with Hyposensitivity subscale score. There was a negative correlation between separation anxiety score and Hyposensitivity subscale score ($r = -0.23$, $P < 0.03$).

None of the independent variables were associated with the PTPD subscale score in general linear model analyses.

Table 2
General linear model analysis for prediction of Tactile Hyposensitivity score

| Independent variables | df | F | Sig. |
|---|---|---|---|
| Separation anxiety score | 1 | 4.242 | 0.045 |
| Oppositional defiant behavior score | 1 | 0.381 | 0.540 |
| Age | 1 | 0.794 | 0.377 |
| Father's age | 1 | 0.762 | 0.387 |
| Father's educational level | 1 | 1.010 | 0.320 |
| Age of mother | 1 | 0.154 | 0.697 |
| Educational level of mother | 1 | 0.183 | 0.671 |
| ADHD type | 3 | 1.778 | 0.164 |
| Gender | 1 | 0.105 | 0.747 |
| Interaction of ADHD type and gender | 3 | 1.147 | 0.340 |

R Squared = 0.464 (Adjusted R Squared = 0.304).

## 4. Discussion

There was not found any published study that surveyed association of the tactile sensory function in different subtypes of ADHD, co-morbidity with ODD symptoms, and separation anxiety symptoms to compare current results with it. Number of ODD symptoms was in association with Hypersensitivity subscale score. It is one of the most striking findings of this study. Previous study reported that higher levels of SMD among children with ADHD were related to greater levels of aggression and psychopathology or emotional and behavior problems [5]. The association suggests that ADHD children with ODD symptoms are different with at least one aspect of tactile perception and have no more significant difficulty with other aspects of tactile perception. It had not been reported before. All of the previous studies usually surveyed only tactile sensitivity and did not separate the Hyposensitivity and hypersensitivity [5].

In the current study, girls were no more than the boys impaired in Hypersensitivity aspect. It is in contrast with a recently and the only found published study about sex differences in tactile defensiveness (TD) in children with ADHD [17]. They reported that females with ADHD display higher levels of TD than males with ADHD. Of course, in their self-report study, all of their ADHD children were combined type. While, the rate of ADHD-combined type in the current study was about 57%. Furthermore, some of their subjects were undertaking stimulants or non-stimulants medications. There is a report that methylphenidate alters sensory processing [19]. Current results are in accordance with a study on twins, reported that mean TD score was not different between males and females [13].

Different types of tactile sensory dysfunction mean scores were not different between the three types of ADHD. However, a study showed that the sensory functioning of a community sample of 49 preschool children with ADHD was impaired. The impairment was not only result of the attention deficits [1]. These results are in agreement with studies that subtypes of ADHD are not different in terms of co-morbid psychiatric disorders [20]. It might be concluded that different types of ADHD are not distinct disorders regarding to tactile sensory function.

Separation anxiety score was not predictor of Hypersensitivity subscale score. It is not in accordance with the report that TD correlates with a fearful temperament and anxiety scores [13]. Previous studies suggested that TD is an 'anxious' response to certain tactile situations which is expressed in avoidance of non-noxious touch and stimuli. In other words, those stimulus are interpreted as threatening [17,18]. However, hyposensitivity aspect of the ADHD children was in association with number of separation anxiety symptoms.

There is a maturational lag in cortical development of brain activity of children with ADHD [21,22]. It is most prominent in the lateral prefrontal cortex which is a host of cognitive functions, such as the ability to suppress inappropriate responses and thoughts [23]. Further studies would show if a more maturational lag is in association with numbers of ODD symptoms and Hypersensitivity dysfunction.

There are some points that should be noticed. First, sensory dysfunction might vary day-to-day. This variation is due in part to the influence of environment on sensory systems. In addition, sensory systems are under complex regulation both from the peripheral receptor to the central nervous system and from central nervous system to brainstem [24].

Second, this study is a cross sectional and does not survey any cause and effect relationship. The checklist is just a screening guide. More, there are other possible interpretations for the results such as informant bias or misunderstanding. It is possible and plausible for parents to misunderstand some of the sensory items. Current results suggest that some problems might be present in tactile sensory functioning in a proportion of children with ADHD. It may point to different abnormalities in the neurological substrate underlying ADHD, ODD and somatosensory function.

There are some other limitations that should be noticed. Intelligence was not assessed formally. Lack of subjective and validated methods for identifying different aspects of sensory integrative dysfunction is a limitation that should be addressed in further studies. Somatosensory function was based on the parental report. It is unknown if this method of assessment is actually providing subjective aspects of somatosensory perception. So, it is clear that these results should be considered as preliminary and further researches should be conducted before form firm conclusions. Future studies should include an external validation such as teachers' report or objective and subjective measures of sensory problem.

## Acknowledgment

The author thanks Drs Alavi, Negarestani, Meftagh, Shokrpour and Chitsaz for their help.

## Appendix: The items and subscales of the tactile somatosensory dysfunction checklist

| **HYPERSENSITIVITY TO TOUCH (tactile defensiveness):** |
|---|
| even gentle or unexpected touch make him/her aggressive, or frightened or worried |
| doesn't like to be hugged by others |
| it seems that he/she avoids or fears of standing near other people |
| if somebody touches him/her from behind, he/she gets afraid |
| doesn't like brush his/her hair or easily annoyed by it |
| oppose friendly touch even with his/her parents |
| doesn't like to be kissed by others |
| react excessively to small cuts, or bite |
| usually keep away or avoid to touch some textures of materials like blanket or rug |
| usually avoid wearing new or tough clothes, jeans |
| try not to use his/her hands while playing |
| he/she is very sensitive to tickling |
| prefer to wear short or shirt with short sleeves or even prefer to wear less clothes or stay unclothed |
| distress to wash his/her face |
| dislike cutting his/her hair, fingernails, or toenails |
| **HYPOSENSITIVITY TO TOUCH (under-responsive):** |
| may not be aware that he/she has been touched, except he/she has been touched forcefully |
| might not be aware that his/her face or hands are dirty or even his/her running nose |
| may hurt himself/herself by biting or pinching |
| put objects into his mouth frequently |
| recurrently hurts other children or animals while playing |
| likes to touch tough and rough objects or surfaces |
| enjoy messy plays with such as soil or glue |
| enjoys touching vibration of mobile |
| **POOR TACTILE PERCEPTION AND DISCRIMINATION:** |
| he/she has trouble in doing fine motor tasks. e.g. buttoning or zipping. |
| he/she wears cloths messy and chaotic. foe example, his/her shoes may be untied. |
| is not able to use scissor well or has trouble in using spoons, forks, or crayons. |
| it is hard for him/her to realize physical quality of objects such as its shape, weight, size, etc. |
